# Whole-Body Diffusion-Weighted Imaging in Chronic Recurrent Multifocal Osteomyelitis in Children

**DOI:** 10.1371/journal.pone.0147523

**Published:** 2016-01-22

**Authors:** Nadine Leclair, Gregor Thörmer, Ina Sorge, Lutz Ritter, Volker Schuster, Franz Wolfgang Hirsch

**Affiliations:** 1 Department of Paediatric Radiology, Leipzig University Hospital, Leipzig, Germany; 2 Siemens Healthcare GmbH, Diagnostic Imaging, Magnetic Resonance Imaging, Erlangen, Germany; 3 Department of Paediatric Rheumatology, Leipzig University Hospital, Leipzig, Germany; Shenzhen institutes of advanced technology, CHINA

## Abstract

**Objective:**

Chronic recurrent multifocal osteomyelitis/ chronic non-bacterial osteomyelitis (CRMO/ CNO) is a rare auto-inflammatory disease and typically manifests in terms of musculoskeletal pain. Because of a high frequency of musculoskeletal disorders in children/ adolescents, it can be quite challenging to distinguish CRMO/ CNO from nonspecific musculosketetal pain or from malignancies. The purpose of this study was to evaluate the visibility of CRMO lesions in a whole-body diffusion-weighted imaging (WB-DWI) technique and its potential clinical value to better characterize MR-visible lesions.

**Material and Methods:**

Whole-body imaging at 3T was performed in 16 patients (average: 13 years) with confirmed CRMO. The protocol included 2D Short Tau Inversion Recovery (STIR) imaging in coronal and axial orientation as well as diffusion-weighted imaging in axial orientation. Visibility of lesions in DWI and STIR was evaluated by two readers in consensus. The apparent diffusion coefficient (ADC) was measured for every lesion and corresponding reference locations.

**Results:**

A total of 33 lesions (on average 2 per patient) visible in STIR and DWI images (b = 800 s/mm^2^ and ADC maps) were included, predominantly located in the long bones. With a mean value of 1283 mm^2^/s in lesions, the ADC was significantly higher than in corresponding reference regions (782 mm^2^/s). By calculating the ratio (lesion to reference), 82% of all lesions showed a relative signal increase of 10% or higher and 76% (25 lesions) showed a signal increase of more than 15%. The median relative signal increase was 69%.

**Conclusion:**

This study shows that WB-DWI can be reliably performed in children at 3T and predominantly, the ADC values were substantially elevated in CRMO lesions. WB-DWI in conjunction with clinical data is seen as a promising technique to distinguish benign inflammatory processes (in terms of increased ADC values) from particular malignancies.

## Introduction

Chronic recurrent multifocal osteomyelitis / chronic non-bacterial osteomyelitis (CRMO/ CNO) is a rare, auto-inflammatory, non-bacterial disease and was first described by Giedion et al. in 1972 [1]. The annual incidence is currently estimated at 0.4 per 100 000 in Germany [2]. To date, etiology and the pathogenesis of CRMO/ CNO are still unknown [3] and there is no specific test or examination for the disease [4]. Because of a high frequency of musculoskeletal disorders in children/ adolescents, it can be quite challenging to distinguish CRMO/ CNO from nonspecific musculosketetal pain or from malignancies.

Initial clinical symptoms of CRMO/ CNO typically are swelling and severe pain of the affected bones [2], while multifocality of bone involvement often only occurs during following episodes of the disease [5–9]. Since clinical symptoms on onset widely overlap with the symptoms of malignant bone tumors, e.g. Ewing’s sarkoma, imaging can play an essential role to exclude malignancy. Furthermore, imaging can help to characterize the disease and to triage patients to adequate treatment. If diagnosed earlier, complications of CRMO/ CNO (e.g. fracture, vertebra plana) may be prevented.

Compared to other whole body imaging tools like PET or bone scintigraphy [3,5–11], whole-body MRI (WB-MRI) has been found to be of superior sensitivity in detecting multifocal edematous lesions, which appear as focal or diffuse hyper-intense regions in fat-saturated T2-weighted images [12]. However, this appearance is non-specific and malignancy can sometimes not be completely ruled out—in uncertain cases, especially in case of unifocal lesions, biopsy remains mandatory to date.

In this regard, diffusion-weighted imaging has been proposed as a potential means for differential diagnosis of inflammatory vs. malignant etiology [11]. Diffusion-weighted imaging (DWI) measures the self-diffusion of water molecules and can be quantified in terms of the apparent diffusion coefficient (ADC), which helps to gain information about tissue cellularity [13]. Malignant lesions often tend to have an increased cellular density which impairs water diffusion and are therefore associated with diminished ADC while edematous bone-marrow presents with a high fraction of free inter-cellular water, resulting in an increased diffusion coefficient.

Nonetheless, studies investigating the clinical value of DWI in children with non-tumorous lesions / in children with CRMO/ CNO are still rare and only involved locoregional imaging of diffusion properties [11]. This may be attributed to the fact that technical constraints have impaired the widespread application of the technique throughout all body regions [14]. Targeted MR imaging, however, cannot be used to assess multifocality. Due to the ongoing evolution of DWI into a whole-body method (WB-DWI), the present study aims to evaluate the method for a systemic investigation of bone-marrow lesions. Our aims were to describe the appearance of CRMO/ CNO in WB-DWI and to evaluate its potential in the classification of the detected lesions.

## Material and Methods

### Patients

The study was approved by the local ethics committee of the Medical Faculty of the University of Leipzig, written informed consent was obtained from the legal guardians of all patients. The use of anonymized data for research purposes is included in the treatment contract between patients and our university hospital. All examinations were conducted between March 2012 –February 2014 in children with known CRMO/ CNO, to rule out new suspected lesions or to reevaluate bone marrow edema as a follow-up. In accordance with the classification used by Fritz et al. [6], initial diagnosis was based on the presence of multifocal, high-signal intensities in the bone marrow in STIR series in corroboration with clinical findings (good general health, relatively mild inflammatory syndrome, moderately elevated erythrocyte sedimentation rate). In case of inconclusive findings, initial diagnosis was made by biopsy.

16 patients (age range 8–17 years; mean age 12.9 years) were included in the study; 11 were female, 5 were male.

The time between initial diagnosis and MR examination varied substantially. All included patients received treatment with non-steroidal anti-inflammatory drugs (NSAID), sometimes for many months before the MR examination included here took place. One patient received treatment with methotrexate and biologicals as well. For further clinical information please see Table 1.

**Table 1:** Clinical baseline data of the included patients. Abbreviations: m (male); f (female); NSAID (non-steroidal anti-inflammatory drugs); Y (Yes); N (No).

Sex	Age on onset	Age (MR Exam)	Time between onset and follow-up MRI (months)	Medication	Biopsy
m	13	15	26	NSAID	Y
f	10	12	28	NSAID	Y
f	10	12	32	NSAID	Y
f	9	15	71	NSAID	N
m	14	16	23	NSAID	N
f	8	8	1	NSAID	Y
f	9	9	1	NSAID	N
f	11	12	15	NSAID	N
f	12	13	15	NSAID	N
m	12	12	2	NSAID	N
f	8	17	19	NSAID	N
f	14	14	11	NSAID	Y
f	12	12	9	NSAID	N
m	10	10	3	NSAID	N
m	11	16	65	NSAID, Methotrexate, Biologicals	Y
f	9	12	47	NSAID	Y

### MRI examinations

Whole-body measurements were performed in a 3T MR scanner (MAGNETOM Tim Trio, Siemens Healthcare, Erlangen, Germany) equipped with a multi stational moving table option, using a multichannel surface coil system (Total Imaging Matrix) and employing parallel imaging technology (iPAT). Thoracic imaging was breath-triggered using a breathing-belt while patients breathed spontaneously. The entire examination, including patient preparation, positioning and scanning took approximately 60–70 minutes in total to complete.

In our standard scan protocol we used axial and coronal 2D short Tau inversion recovery (STIR) images and axial DWI. Imaging parameters for STIR were as follows: Repetition Time / Echo Time (TR/TE) = 4300–4600 ms / 49 ms; Inversion Time (TI) = 220 ms, flip angle = 150°, Field of View (FoV) = (500x500) mm, Matrix (MX) = 448x336, Voxel Size = 1.1 x 1.5 mm, Slice Thickness = 5 mm, 1 average. We used inversion recovery because it enables a more robust fat suppression over large fields-of-view than spectral fat suppression techniques.

Axial isotropic diffusion-weighted images (DWI) from head to feet were acquired using b-values of 50 s/mm^2^ and of 800 s/mm^2^, with a slice thickness of 4 mm. Whole-body coverage was usually achieved in 6–8 continuous stations, dependent on the height of the child, using a free-breathing technique in the thoracic station. Depending on the number of deployed stations, DWI scanning took an extra of 12–16 minutes. Scanning parameters for diffusion weighted series were TR 6100–7200 ms, TE 62 ms, flip angle 90°, fatsat, bandwidth 976 Hz/ pixel, FoV = (500x500) mm, MX = 256x256, Voxel Size = 1.95 x 1.95 mm, 1 average.

The b = 800 s/mm^2^ images were composed and reconstructed in the coronal plane (5 mm) and as thick 3D maximum intensity projections (MIPs), which were displayed using an inverted grey scale for a PET-like image resemblance using the standard inversion feature of the syngo software. ADC maps were generated automatically with the scanner software, using mono-exponential fitting.

### Image interpretation

Image interpretation was performed by using a postprocessing workstation (syngo, Siemens Healthcare). Images were evaluated for the presence of focal, high-signal intensity in STIR series both in axial and coronal views. In accordance to the found positions, b800-images were evaluated for respective signal alterations. Circular regions of interest (ROIs) were placed in bone marrow edema and normal tissue on the opposite, normal appearing side (reference region) to determine the mean ADC (± standard deviation) value for each location. Size of the ROI was individually adapted to the actual lesion size but lesions with a diameter of less than 5 mm were excluded. This ROI was then copied and subjectively shifted to the corresponding reference position on the opposite side.

All examinations were evaluated by two readers in consensus. Furthermore, image quality of DWI scans was rated by applying a 3-point scoring system (0 = non diagnostic; 1 = impaired quality; 2 = good quality) intending to rule out non-diagnostic scans. The readers had access to all clinical patient information including age, sex, family history and painful sites.

## Results

In whole-body STIR sequences, a total of 38 lesions were detected in our cohort of 16 patients, resulting in 2 lesions per patient on average. The most commonly affected regions in our patient cohort were the pelvis/ hip and the lower limbs (see Table 2).

**Table 2:** Location of signal alterations in STIR and DWI (here, only the 25 lesions with a relative ADC increase of more than 15% were considered).

Localization	STIR	DWI
Neurocranium	-	-
Clavicula/ Shouldergirdle	1	1
Humerus	3	3
Radius / Ulna	-	-
Spine	1	1
Pelvis / Hip	15	10
Femur	3	2
Tibia / Fibula	9	7
Feet	1	1

**Fig 1:**
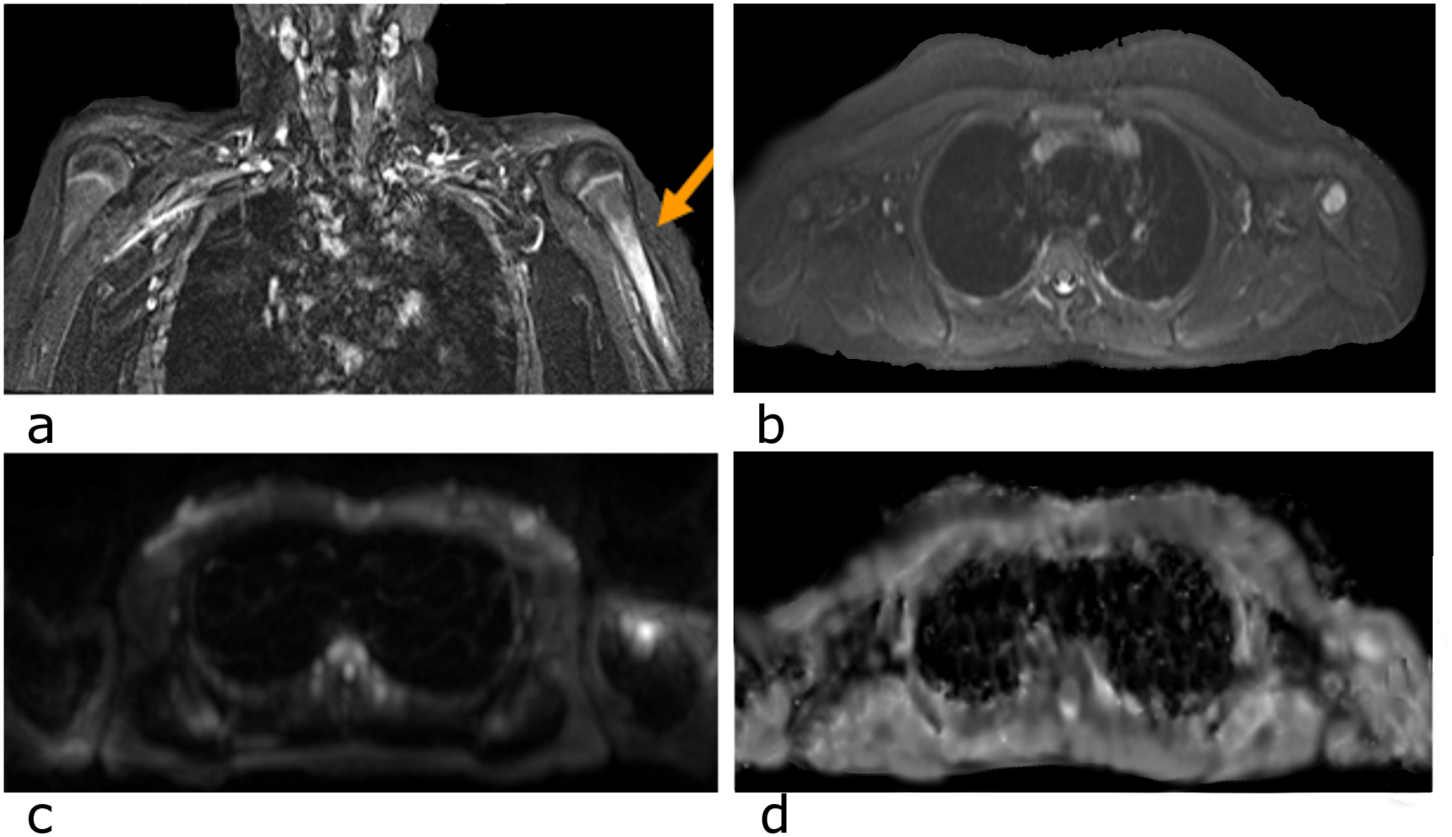
MR images of 12-year old girl with known CRMO. a) STIR-weighted images show diaphyseal edematous bone marrow involvement, periostal reaction of the left humerus b) axial series showing large and homogenous bony involvement c) signal alteration in DWI b = 800 images d) ADC increase at the corresponding site.

Most lesions appeared as asymmetric, ill- and irregular- defined changes in the bone marrow with a hyperintense appearance in STIR images. Foci were typically settled in metaphyseal and epiphyseal regions adjacent to a growth plate of tubular bones, as well as in the sacrum, the spine and the pelvis. There was one case of diaphyseal involvement, representing as a huge lesion with periostal reaction of the left humerus (Fig 1).

### DWI

In 2 patients DWI could not be evaluated because of non-diagnostic image quality. Sources of image quality degradation were motion and ghosting artifacts. In the remaining 14 patients, 33 lesions were found in DWI which showed corresponding alterations in STIR and were included in the evaluation. Of these, 25 showed signal alterations in DWI higher than 15% compared to the contralateral side (Table 3). Lesions seen only in DWI with no matching findings in STIR were excluded from the evaluation.

**Table 3:** Measured ADC value for lesion, reference regions, the corresponding ratio and location. Abbreviations: Y (Yes); N (No).

#	ADC (lesion)	ADC (reference region)	ADC ratio	Localization	Symptoms (Pain)
1	1754 ± 442	877 ± 508	2.00	Right clavicula	Y
2	1241 ± 307	707 ± 369	1.76	Left humerus	N
3	1849 ±144	853 ± 197	2.17	Left tibia	Y
4	1513 ± 119	1194 ± 274	1.27	Right tibia	N
5	497 ± 125	421 ± 77	1.18	Right pelvis	N
6	975 ± 91	577 ± 268	1.69	Left humerus	N
7	884 ± 95	587 ± 238	1.51	Right sacrum	Y
8	655 ±121	610 ± 71	1.07	Right pelvis	N
9	987 ± 255	449 ± 239	2.20	Right foot	Y
10	1578 ± 54	559± 115	2.82	Left acetabulum	N
11	1272 ± 190	580 ± 44	2.19	Right pelvis	Y
12	1528 ± 76	767 ± 221	1.99	Left pelvis	N
13	1672 ± 42	402 ± 215	4.16	Right tibia	Y
14	1449 ± 218	1112 ± 160	1.30	Left hip	Y
15	1755 ± 125	477 ± 185	3.68	Right pelvis	Y
16	918 ± 173	460 ± 209	2.00	Right hip	N
17	1623 ± 498	1663 ± 315	0.98	Left tibia	N
18	1569 ± 252	752 ± 247	2.09	Left humerus	Y
19	832 ± 47	620 ± 80	1.34	Left sacrum	N
20	768 ± 58	532 ± 61	1.44	Right sacrum	N
21	1340 ± 367	1190 ± 584	1.13	Right tibia	N
22	1663 ± 138	363 ± 262	4.58	Right sacrum	Y
23	1682 ± 160	598 ± 41	2.81	Left femur	N
24	1427 ± 12	692 ± 43	2.06	Right femur	N
25	891 ± 92	794 ± 121	1.12	Right pelvis	N
26	1640 ± 144	715 ± 522	2.29	Right tibia	N
27	1680 ± 141	863 ± 262	1.95	Lumbar vertebra 2	Y
28	1642 ± 165	1648 ± 266	1.00	Left knee	N
29	949 ± 108	1198 ± 123	0.79	Right tibia	N
30	1193 ± 70	940 ± 142	1.27	Left tibia	N
31	709 ± 63	720 ± 94	0.98	Right pelvis	N
32	1197 ± 137	908 ± 114	1.32	Left tibia	N
33	1035 ± 153	1006 ± 104	1.03	Right pelvis	N

**Fig 2:**
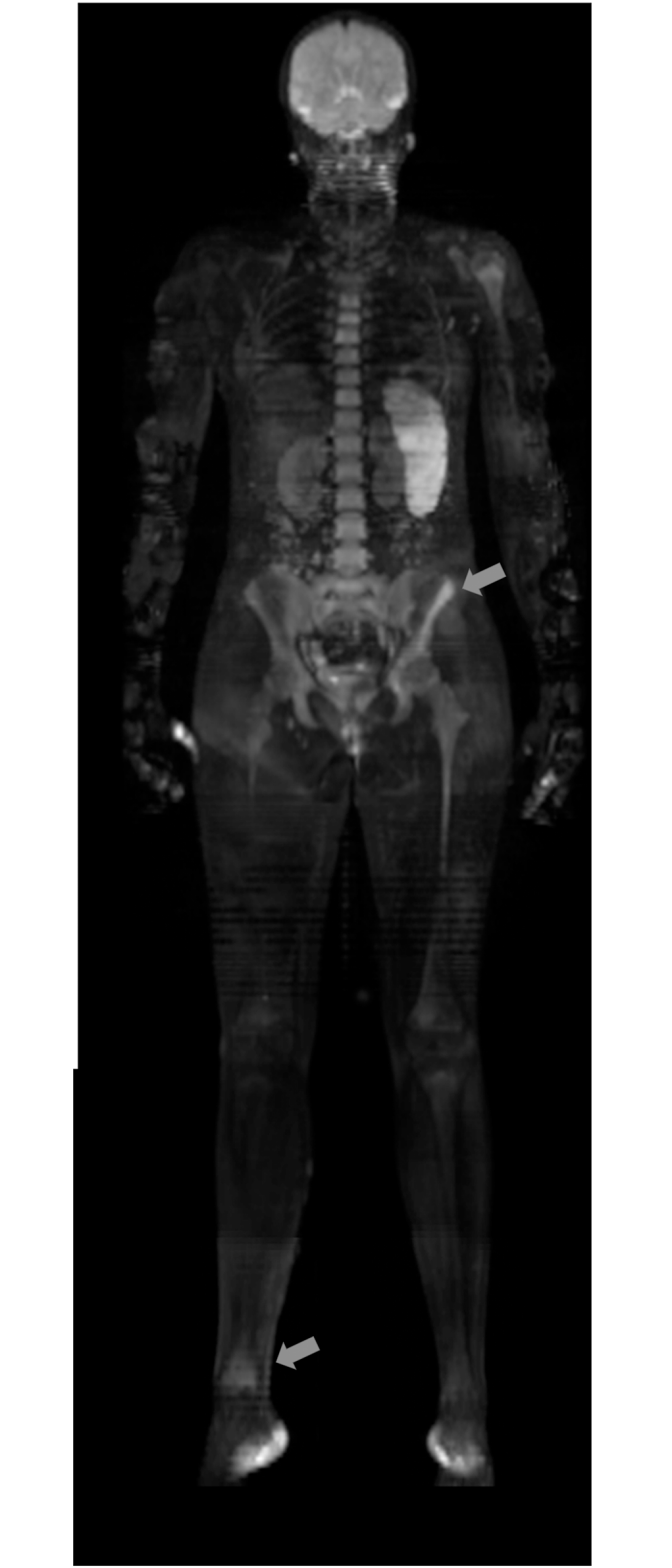
“At-a-glance” visualisation of Whole-body-DWI. Intuitive visualisation of Whole-body-DWI with maximum intensity projection MIP in a 12-year-old girl with multifocal CRMO manifestations shows signal alterations on DWI in the left pelvis (Os ilium) and in the right ankle/ distal tibia.

**Fig 3:**
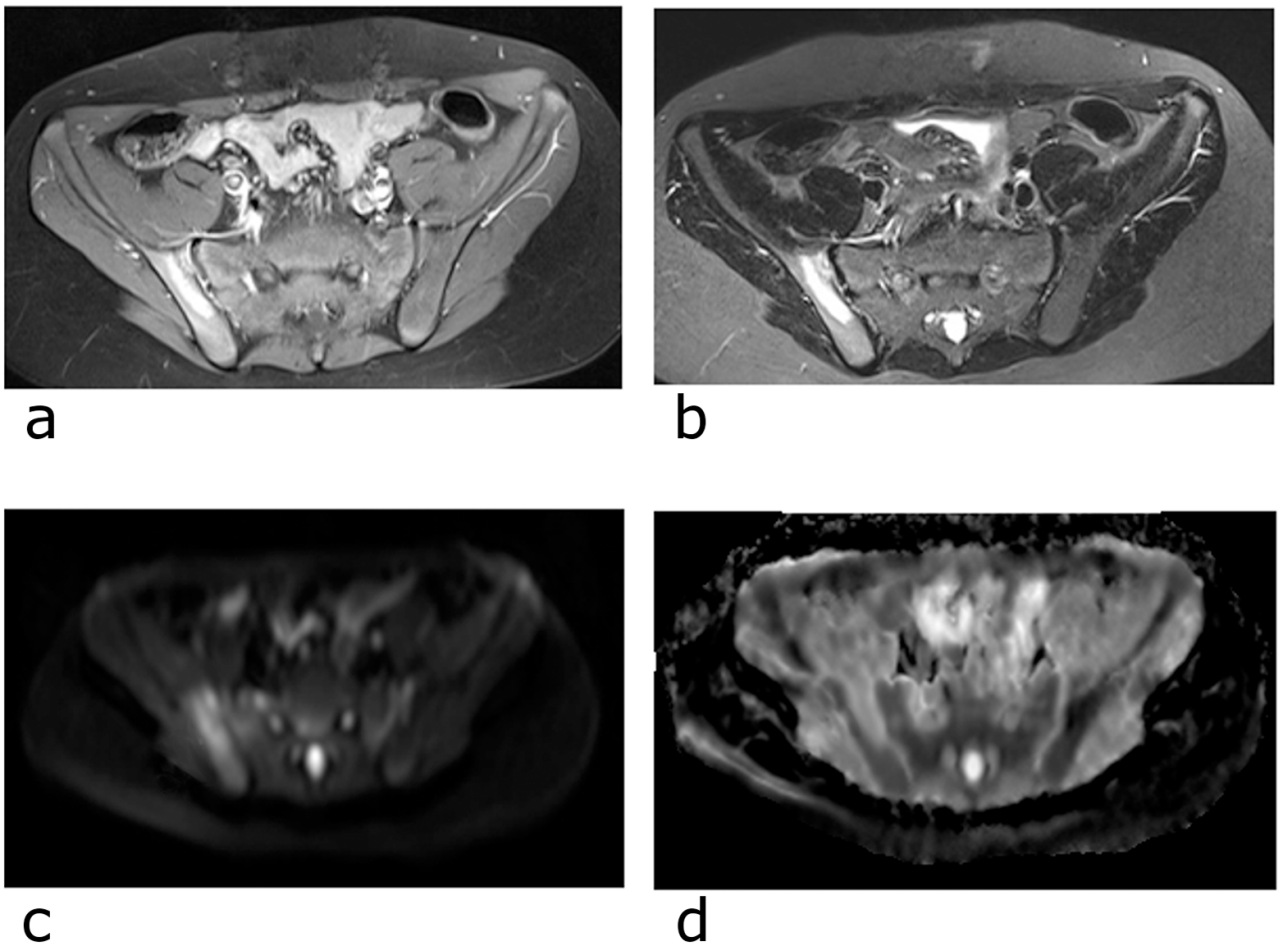
Multiparametric MRI. a) contrast-enhancement of a CRMO-lesion in the right Os ilium on axial T1-TSE FS b) corresponding signal alteration on STIR images c) correlating signal changes on axial DWI b800 d) and high signal intensity on ADC.

**Fig 4:**
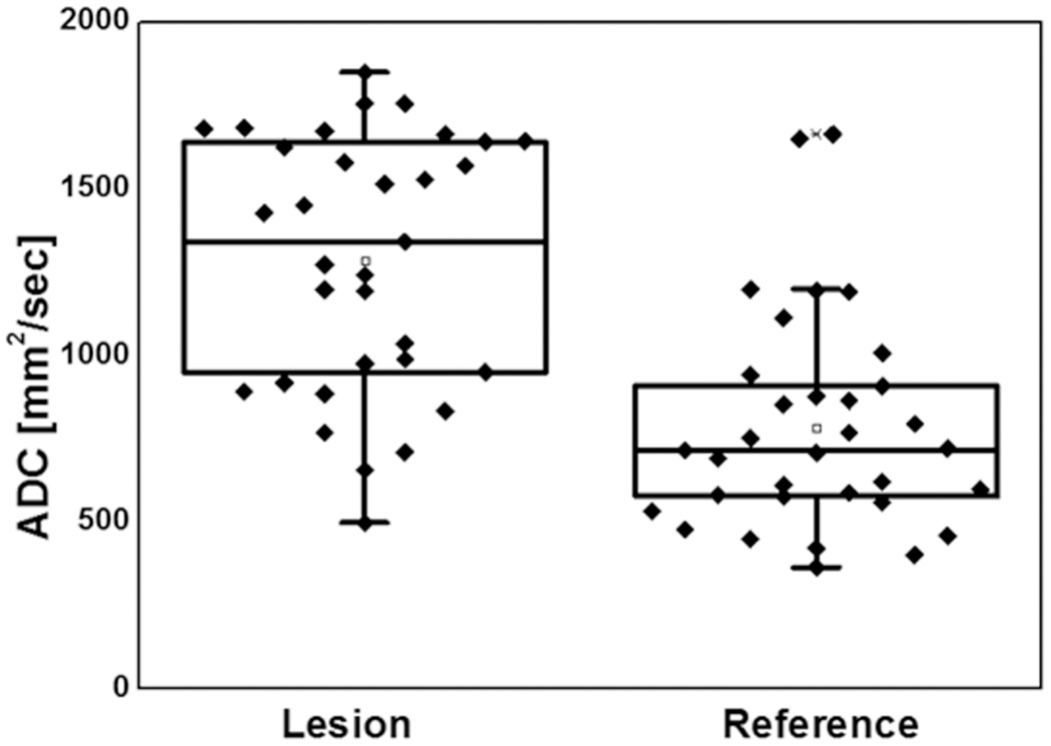
Box-Plot of ADC values. Increased ADC in CRMO-lesions compared to the corresponding reference regions. Since bone marrow cellularity is highly dependent form lesion location and other individual clinical factors, absolute ADC values both in affected and normal bone varied substantially, which made the recommendation of a generalized threshold impossible.

Skeletal edema detected in STIR images showed up as focal or diffuse area of high-signal intensity on high b-value and also a high ADC-value in WB-DWI (Figs 1–3) in most of our cases. In comparing the apparent diffusion coefficient value of the signal alteration site and the values of the corresponding opposite/ healthy side, one can detect that the apparent diffusion coefficient value of the lesions are relatively higher compared to the healthy side (Table 3). The mean ADC value in lesions was 1283 mm^2^/sec, and 782 mm^2^/sec in corresponding reference regions (Fig 4). A paired *t* test revealed a statistically highly significant (p<0.0001) difference between lesion and reference values (t = 6.638, sample size 32). By calculating the ratio (lesion to contralateral; see also Table 3), 82% of all lesions showed a relative signal increase of 10% or higher and 76% showed a signal increase of more than 15%. The median relative signal increase was 69%.

## Discussion

The clinical potential of DWI in children with suspected CRMO/ CNO is to further characterize tissue in terms of biologic tissue characteristics rather than morphologic appearance and is a promising measure to confirm the benignity of multifocal lesions in these children.

While previous studies already have shown the clinical utility of DWI for specific body regions in mixed cohorts of children with different benign-inflammatory and malignant diseases, this study is the first to implement and assess DWI as a whole-body technique in a cohort of children with the rare auto-inflammatory disease CRMO/ CNO.

From a technical point of view, WB-DWI can be reliably performed in children. The examination itself requires an extra scan time of approximately 15 minutes. Even though the youngest patient was only 8 years old, none of the patients cancelled the examination due to positioning discomfort or anxiety because of gradient-associated vibrations and sound pressure during diffusion-weighted scanning. However, the authors believe that it is an essential prerequisite to perceptively prepare infant patients before the whole-body scan. When reading WB-DWI series, some general technical aspects should be kept in mind: the detection of skeletal edema on WB-DWI may be affected in areas of movement such as the anterior ribs and sternum [14]. Visibility of skull vault infiltrations can be impaired because of the adjacent high signal of the normal brain and the visibility of skull base disease is impaired because of susceptibility effects [14]. In our small cohort, no lesion was found in the skull base or in the neurocranium. Furthermore, magnetic field inhomogeneties secondary to metal and air interfaces (for example bracelets) may induce artifacts that cause false positive or false negative DWI lesions and at the same time can conceal lesions in the adjacent bones. However, when considering WB-DWI as an additional examination that is read in association with conventional morphological WB-MRI sequences (e.g. STIR), false-positive findings can be easily ruled out. Notwithstanding the non-diagnostic quality of WB-DWI examination in two cases due to severe ghosting artifacts and motion—without any identifiable shortcoming regarding the conduction of the scans—a technical success rate of 90% was considered acceptable/sufficient. Upcoming techniques such as slice-dependent shim [15] have the potential to overcome these limitations in the near future.

Clinically, DWI has been already shown to be useful to detect and to further characterize inflammatory bone marrow lesions in terms of relative changes of cellularity. With growing water content and increasing grade of inflammation, edematous bone marrow returns higher signal intensities in STIR sequences and higher ADC values [16–19]. One major challenge in interpretation of MR scans of the infant skeleton, however, is the signal appearance of bones in general: The normal adolescent and infant bone marrow pattern shows high signal intensity in STIR weighted images, especially near the epiphyses and high signal intensities are also seen in DWI. Conditions that make it more difficult to interpret WB-MRI in children than in adults.

As it is depicted by the presented results, edematous lesions of the bone marrow tend to appear as clear and distinct areas of signal alteration in DWI. Since the ability to detect bone marrow lesions is dependent on the signal intensity of the background bone marrow and surrounding tissues, the superior lesion conspicuity compared to conventional STIR imaging may be attributed to the faint anatomical background in DWI [11]. Referring to the multifocal nature of CRMO, the technique may even contribute to diagnosis in terms of easier lesion detection throughout the body. In unifocal lesions, WB-DWI in conjunction with clinical data is seen as a promising technique which may help to distinguish benign inflammatory processes (in terms of increased ADC values) from early manifestation of particular malignancies. However, in this study we only included lesions with matching findings in DWI and STIR. Lesions only visible in DWI were deliberately excluded, since the aim of this study was to evaluate the potential of DWI to better characterize lesions detected with conventional WB imaging and not to compare the sensitivity of both parameters. This could be the topic of further investigation.

Previous studies have shown that osteoporotic caused vertebral fractures and fractures caused by malignant lesions differ significantly when it comes to ADC values, potentially allowing for a differential diagnosis of benign and malignant entities [13]. In comparison to bone tumors, which come along with restricted diffusion appearance and a consecutively diminished ADC value [20], benign inflammatory changes seem to dispose a diffusion alteration which comes along with corresponding high ADC values [16] in most cases. Based on the presented results, this seems to count for bony changes as well: even though lacking a control group of patients with malignant changes for comparative purposes, most of all lesions showed increased ADC values compared to respective reference regions. This seems to be in good accordance with previous studies, which have investigated musculoskeletal, locoregional DWI in rheumatological issues [11,21].

However, the relation of bone marrow cellularity and ADC values is highly dependent on the water, cellular and fat volume of the marrow. Its conversion status is reliant on patient`s age, gender, the anatomic location and even the treatment status [16]. The relationship in between these components is non-linear and varies significantly through the different body regions [16]. For example, (intermediate) high signal intensity is seen normally in the appendicular skeleton, i.e. the femura, marking (mixed) red bone marrow [16]. Accordingly, our results reveal a substantial variation of the absolute ADC values both in affected and normal bone. Furtherrmore, it is commonly known that imaging parameters as well as the imaging hardware may generally affect the results of ADC calculation [22]. To the current point in time this makes the independent recommendation of an ADC threshold difficult, if not impossible. The calculation of an ADC ratio, as proposed here, needs further evaluation but might overcome the mentioned limitations of absolute ADC measurements.

This work had several limitations. First, the number of patients was very limited. This is due to the fact that it was decided to preserve a homogenous study cohort of CRMO patients only. Previous studies also suffered from the rare incidence of the disease, with cohorts typically ranging between 9 and 21 patients [3,6,8]. Second, some of the patients had a long history and received treatment over years. This could be also a reason for the relatively rare bone marrow affection in some of our patients, compared to the literature. Furthermore, discrepancies between the number of lesions visible with STIR and DWI may be attributed to effects of treatment, since effects on the cellular level appear much earlier. In one case, for example, the patient was treated with NSAID, methotrexate and biologicals and was asymptomatic at the time of follow-up MRI. Accordingly, no “active” lesions in DWI have been found. In an optimal study setting, WB-DWI would have been performed in a population of newly diagnosed and/ or untreated patients. Third, WB-DWI at a magnetic field strength of 3T remains challenging because of less optimal fat suppression and increasing susceptibility artifacts compared to 1.5T and may have impaired the overall technical success rate. Therefore reading was performed in consensus, which is a further limitation of this study. Last, no further contrasts than STIR, i.e. T1w contrast-enhanced scans, were acquired in order to keep the follow-up exams non-invasive. In the setting of follow-up studies/ established CRMO, we actually think that contrast-enhancing studies are dispensable in general.

However, three out of our patients underwent T1w contrast-enhanced MR examinations less than a week before the WB-scans. The reported, enhancing areas in T1-weighted fat-sat contrast-enhanced series corresponded almost perfectly with alterations in the diffusion-weighted series.

In conclusion, WB-DWI can be reliably performed in children at 3T and predominantly, the ADC values were substantially elevated in CRMO lesions. WB-DWI in conjunction with clinical data is seen as a promising technique to distinguish benign inflammatory processes (in terms of increased ADC values) from particular malignancies.
